# Emotion-Motion Interactions in Conversion Disorder: An fMRI Study

**DOI:** 10.1371/journal.pone.0123273

**Published:** 2015-04-10

**Authors:** Selma Aybek, Timothy R. Nicholson, Owen O’Daly, Fernando Zelaya, Richard A. Kanaan, Anthony S. David

**Affiliations:** 1 Section of Cognitive Neuropsychiatry, King’s College London, Institute of Psychiatry, London, SE5 8AF, United Kingdom; 2 Laboratory for Behavioral Neurology and Imaging of Cognition, Fundamental Neurosciences, Geneva University, Rue Michel-Servet 1, 1211, Genève, Switzerland; 3 Department of Neuroimaging, King’s College London, Institute of Psychiatry, London, SE5 8AF, United Kingdom; 4 Department of Psychiatry, University of Melbourne, Austin Health, Heidelberg, VIC, 3084, Australia; Vanderbilt University, UNITED STATES

## Abstract

**Objectives:**

To evaluate the neural correlates of implicit processing of negative emotions in motor conversion disorder (CD) patients.

**Methods:**

An event related fMRI task was completed by 12 motor CD patients and 14 matched healthy controls using standardised stimuli of faces with fearful and sad emotional expressions in comparison to faces with neutral expressions. Temporal changes in the sensitivity to stimuli were also modelled and tested in the two groups.

**Results:**

We found increased amygdala activation to negative emotions in CD compared to healthy controls in region of interest analyses, which persisted over time consistent with previous findings using emotional paradigms. Furthermore during whole brain analyses we found significantly increased activation in CD patients in areas involved in the ‘freeze response’ to fear (periaqueductal grey matter), and areas involved in self-awareness and motor control (cingulate gyrus and supplementary motor area).

**Conclusions:**

In contrast to healthy controls, CD patients exhibited increased response amplitude to fearful stimuli over time, suggesting abnormal emotional regulation (failure of habituation / sensitization). Patients with CD also activated midbrain and frontal structures that could reflect an abnormal behavioral-motor response to negative including threatening stimuli. This suggests a mechanism linking emotions to motor dysfunction in CD.

## Introduction

Conversion disorder (CD), formerly called hysteria but now also referred to as functional neurological symptom disorder[1], is the presence of neurological symptoms in the absence of neurological disease. In the case of motor CD, patients typically display weakness (such as a paraplegia) or abnormal movements (such as a tremor or dystonia). The prime aetiological mechanism of CD is presumed to be psychological, but traditional (e.g. Freudian) models which postulate the disorder to be a direct result of psychological stressors have been challenged, as such stressors are not evident in all patients[2]. Contemporary models attempt to link symptoms, cognition and behavioural factors down to the neural level[3], but there are few robust experimental findings which support such models or provide neuroanatomical specificity.

The focus of functional imaging studies to date in CD has largely been to investigate patients with motor symptoms using motor system paradigms. In contrast, we have recently shown abnormal activation in cortical regions during an emotional memory task (recall of stressful events of likely aetiological significance). This included abnormal activation in motor planning (supplementary motor area—SMA) and sensory integration regions (right temporo-parietal junction) as well as frontal lobe emotional control areas in patients with sensori-motor CD[4]. We interpreted these findings as suggesting an interaction between emotion regulation regions and those linked to motor and sensory symptoms. A similar limbic-motor interaction has been shown in CD patients presenting with movement disorders during an implicit emotion task (processing of emotional—fearful and happy—faces) with increased amygdala and SMA functional connectivity along with some evidence of a failure to habituate to emotional stimuli[5]. This is important since it is well established that the amygdala is a key limbic system structure in emotion recognition and in particular identification of threat signals[6,7]. Its connection to motor loops is essential in order to interrupt ongoing motor activities in potentially threatening situations[8]. The amygdala (more specifically the centromedial nuclei) has also been shown to be involved in affective-motor pathways, both in animals and humans, which might mediate complex motor functions such as the startle response or flight reaction[9].

In addition, there has recently been a study of children with CD which found shorter reaction times to negative emotional faces compared to neutral faces consistent with a state of increased vigilance and motor readiness to emotional stimuli[10]. Further evidence of an abnormal state of emotional arousal in CD also comes from findings of elevated basal cortisol levels in the seizure variant of CD[11] that correlate with attentional bias towards threatening stimuli[12].

In the present study we sought to further investigate the processing of negative emotions, we therefore used sad and fearful faces as stimuli. Based on previous data[5], we hypothesized that CD patients would exhibit a pattern of response to negative emotions over time that differs from controls subjects by showing a failure to habituate. As well as the amygdala, we examined other neural systems through which abnormal emotional processing may lead to consequences in the motor system, believed to be the final common path in the pathophysiology of motor CD.

## Methods

### Subjects

Fifteen adult patients with DSM-IV diagnoses of motor CD were recruited from neurology or neuropsychiatry settings in South East London and fourteen age-and-sex matched controls were recruited from primary care clinics in the same area as part of a larger study of CD. Criteria A and D of the DSM-IV definition (the presence of neurological symptoms; these are incompatible with known neurological conditions) were established by certified neurologists based on the clinical history, examination and appropriate investigation. Criterion B was established by a consultant neuropsychiatrist. All participants were selected on the basis of having experienced at least one stressful life event in the year preceding illness onset (or equivalent epoch in controls) and symptom duration of <2 years, as they were part of another fMRI study focussing on preceding stressors[4],[13]. Three patients were removed from the analysis after failing to perform the task satisfactorily (low accuracy of <50% correct trials) and/or because of excessive movement (defined as >3mm in any axis), giving final sample sizes of 12 CD cases (9 female) and 14 controls (11 female)—see Table 1 for details. Non-fluent English speakers, those with a comorbid neurological or psychotic disorder were excluded. The study was approved by a local Research Ethics Committee (Bromley REC 07/H0805/33). After complete description of the study, written informed consent was obtained from all subjects.

**Table 1:** Subjects’ Characteristics.

	CD patients (N = 12)	Healthy controls (N = 14)	Significance
**Mean age (years) (SD)**	34.9 ± 11.9	36.3 ± 8.8	ns^†^ (p = 0.7)
**Female/male**	9/3 (75%)	11/3 (79%)	ns* (p = 0.8)
**Estimated IQ**	104.4 ± 9.7	109.5 ± 12.7	ns^†^ (p = 0.2)
**Depression score (mean)**	8.6 ± 6.4	5.5 ± 4.1	ns^†^ (p = 0.1)
**No. with depression (HADS >11)**	5 (42%)	0 (0%)	p = 0.012*
**Anxiety score (mean)**	12.2 ± 5.6	8.6 ± 4.0	ns^†^ (p = 0.1)
**No. with anxiety (HADS >11)**	7 (58%)	4 (29%)	ns* (p = 0.1)
**Sexual abuse**	3 (25%)	2 (14%)	ns* (p = 0.2)

* Fisher’s exact test

^†^ *Student’s t-test*

### Clinical data

Mood was assessed using the self-report Hospital Anxiety and Depression Scale (HADS)[14] with the following cut-off scores (out of 20): <7 = no depression/anxiety; 8–10 = borderline depression/anxiety; >11 = clinical depression / anxiety. IQ was estimated with the National Reading Test (NART)[15]. Experiences of sexual abuse were obtained as part of a detailed semi-structured clinical interview[16]. See Table 1 for details.

### Affective fMRI task

Subjects viewed affective stimuli using sad and fearful ‘Ekman’ faces[17] in an event-related task described previously[18]. The facial expression stimuli were presented in two separate fMRI series—sad and fearful—and in each series, 40 affective stimuli and 20 neutral faces were presented for 2 seconds in a pseudorandom order. The order of fearful and sad stimulus runs was counterbalanced across subjects Both affective stimuli (sad and fearful) were subdivided in the same scanning session into two groups of 20, each containing morphed expressions calibrated to 50% and 100% ‘affective valence’ (emotion intensity) respectively. To improve the subsequent analysis of over-lapping haemodynamic response functions, the inter-trial interval time was varied using a Poisson distribution with a mean of 5 seconds and a standard deviation of 1.55 seconds.

For each of the stimuli, subjects were asked to determine the gender of the presented face (also pseudo-randomised), to which they responded by means of a button-box, placed on their dominant hand (or less affected hand if upper limb motor symptoms). There were no instructions about or mention of the affective content of the images so the task is therefore ‘implicit’ with respect to emotion processing. Accuracy and response times were recorded and used in the analysis (see below).

### Image acquisition and pre-processing

MRI scans were performed on a GE 3-Tesla Signa HDX scanner, using an 8-channel RF coil. During the task, a temporal series of 180 gradient-recalled, echo-planar imaging (EPI) image-volumes was acquired (TR/TE = 2000/30ms, total: 6:00min); 38 near-axial slices were collected with an isotropic spatial resolution of 3.4mm. A single high resolution, multi-shot, spin echo EPI scan (1.875 x 1.875 x 3.3mm) and a single 3D, T1-weighted Spoiled Gradient Recalled volume with an isotropic resolution of 1.1mm, were acquired for co-registration and spatial normalisation.

Data were processed using Statistical Parametric Mapping (SPM8, http://www.fil.ion.ucl.uk/spm) and adjusted for slice timing, realigned to the first image of the first run, normalized to the Montreal Neurological Institute (MNI) atlas and smoothed using an 8-mm Gaussian kernel. In order to correct for movement artefacts, first-level analyses were carried out using the Robust Weighted Least Squares (RWLS) tool available in SPM8[19].

### Statistical analysis

Demographic and clinical data were compared between groups using Fisher’s Exact Test, and unpaired t–tests. Behavioural data were compared between groups using repeated measures analysis of variance for reaction times and non-parametric tests for accuracy.

### Image analysis

#### First level analyses

In the first level analysis, for each emotion valence, predicted BOLD response to each block was modelled in SPM8 using a vector of onsets derived from the randomly defined time of occurrence of each event, convolved with the haemodynamic response function. As the Neutral condition constituted our baseline, we computed the contrasts of Fear-Neutral and Sad-Neutral (hereafter referred to as the ‘**Emotion model**’). The response time of the subject to each of the stimuli was used as the event duration, as beyond this time continued cognitive engagement with the task is less certain.

In order to evaluate temporal changes in response amplitude, we built an additional first-level model. For each of the conditions (fear, sad and neutral), the ‘temporal-modulation’ option of the first-level model specification in SPM-8 was used, and the ‘1^st^ order Time Modulation’ option was chosen which models the presence of a linear change in stimulus-evoked activation as a function of time. We refer to this model as the ‘**Linear-change model**’ hereafter.

#### Second-level analyses: Region of interest analysis

As we had an a priori hypothesis of differential activation in the amygdala in response to negative emotions[20], we conducted a region of interest (ROI) analysis on bilateral amygdala by using an inclusive anatomical mask obtained from ‘fwupickatlas’ toolbox of SMP-8. We only report small-volume corrected results that survived Family-Wise Error (FWE) correction for multiple comparisons at significance levels of p<0.05 at the peak levels on the ROI. We compared the effects of group (CD vs controls), valence (sad, fear) and interaction group x valence. Both the Emotion and Linear-change first level models were tested.

#### Second-level analyses: Whole brain analysis

To obtain second level within- and between-group z-statistics, statistical maps were thresholded at a z value>3.2 (cluster forming threshold, p<0.001) and a cluster-corrected FWE correction threshold (p<0.05) was calculated using Gaussian random field theory[21]. In the second level, random-effects analysis, we compared the effects of group, valence and group x valence interaction. We only report significant clusters (p<0.05) after FWE correction at the cluster level. Both the Emotion and Linear-change first level models were tested.

#### Second-level analyses: Effect of mood

A greater proportion of subjects with clinically relevant depression scores was found in the patient group, (see Table 1). In order to account for an effect of depression in our imaging analysis, we thus added the HADS depression scores as covariates in all second level analyses (both ROI and whole-brain). Concerning anxiety, no group difference was found for HADS anxiety scores and these were not added as covariates. However, we did repeat the ROI linear-change analysis with anxiety scores, in order to ensure that even a subclinical anxiety ‘state’ could not account for an enhanced and sustained response to negative emotions in particular to fear stimuli. Our results stayed significant and we only report here the data obtained without the anxiety score as a covariate.

## Results

### Subjects’ characteristics

Patients and controls did not differ statistically in terms of age, gender, estimated IQ and history of sexual abuse (Table 1). Mean depression and anxiety scores did not significantly differ between groups, though as noted, the proportion of subjects with HADS depression scores >11 was significantly higher in patients. All the 12 CD patients had symptoms (motor +/- sensory) at time of scanning, with a mean duration of symptoms of 14.2 months.

### Behavioural results

Overall, there were no differences in reaction times between patients with conversion disorder (mean RT in ms (±SD): Neutral: 1046(±127) / Fear: 1027(±147) patients / Sad: 980(±125) and healthy controls (Neutral: 1026 (±165) Fear:1004(±166) / Sad: 998(±131) [(F(1,24) = 0.026, p = 0.87). No main effect of condition (emotional valence) nor interaction was found (all p>0.05).

For the task accuracy (percentage of correct responses), there was no interaction nor main effect of group; patients with conversion disorder correctly identified 96.4% of stimuli and controls 96.9%. There was a significant effect of condition [(F(1,27) = 10.1, p = 0.04) with neutral stimuli generating lower accuracy rates (92.9% ±5.1) then emotional faces, both sad (98.9%±5.4) and fearful(97.4%±5.4), in both groups].

### Imaging results

#### Region of interest analysis

**Fig 1:**
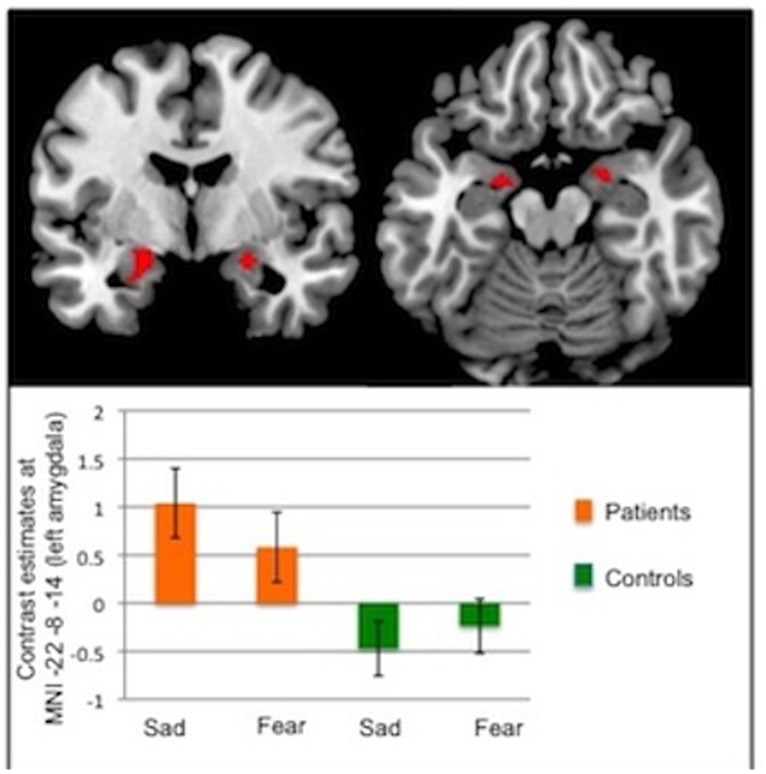
ROI analysis: Group effect in the Emotion Model. *Upper panel*: Activation in the amygdalae in response to emotional faces showing a group effect (CD patents > healthy controls). Statistical maps have been thresholded at p<0.005 uncorrected for display purposes. A significant increased activity was found in the left amygdala (FWEp = 0.027, z = 3.3, [–22–8–14]). Of note, the right amygdala activation displayed here did not reach conventional levels of significance after correction for multiple comparisons (FWE p = 0.078 at MNI [22–4–14]). *Lower panel*: Graph showing contrast estimates for left amygdala activation in response to each negative emotion (sad, fear) separately for the 2 groups: CD patients (in orange) and healthy controls (in green).

In the Emotion model, a significant group effect was found in the left amygdala (p = 0.027, z = 3.3, [–22–8–14]), with enhanced activity for both emotions in CD patients (Fig 1). Similar increases in activation were found in the right amygdala but these did not survive correction for multiple comparisons. There was no effect of valence and no interaction effect.

**Fig 2:**
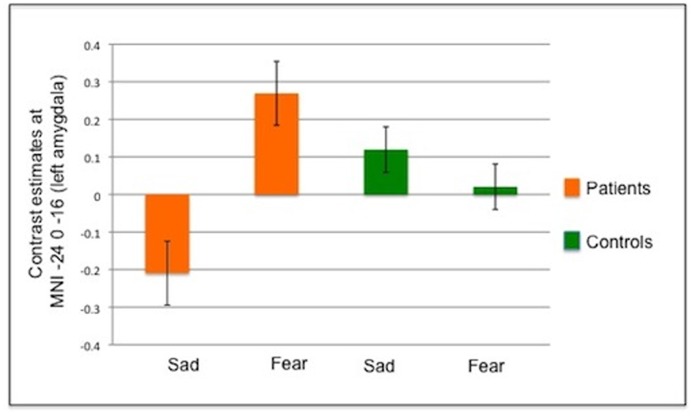
ROI analysis: Interaction effect in the Linear-change Model. Graph showing group-level beta estimates for the linear change in the responsiveness of the left amygdala to the presentation of emotional stimuli. Here a positive beta indicates a linear increase in the magnitude of the response elicited by stimuli following repeated exposure. Negative betas indicate the response amplitude reduced over time (see S1 Fig for a graphical representation of the predicted BOLD time series scaled by these group beta coefficients).

In the Linear-change model, an interaction (valence x group) effect (Fig 2) was found in the left amygdala (p = 0.015, z = 3.6, [–24 0–16]), with CD patients showing enhanced activity to fearful stimuli as a function of time, when compared to sad stimuli, relative to healthy controls. This suggests that patients not only do not habituate to fearful faces but even become sensitized to this repeated stimulus, unlike healthy controls who do not show this increased pattern over time (see Fig 2 lower panel). This result remained significant when the HADS anxiety scores were added as a covariate. No main effect of group or valence was found.

#### Whole-brain analysis

**Fig 3:**
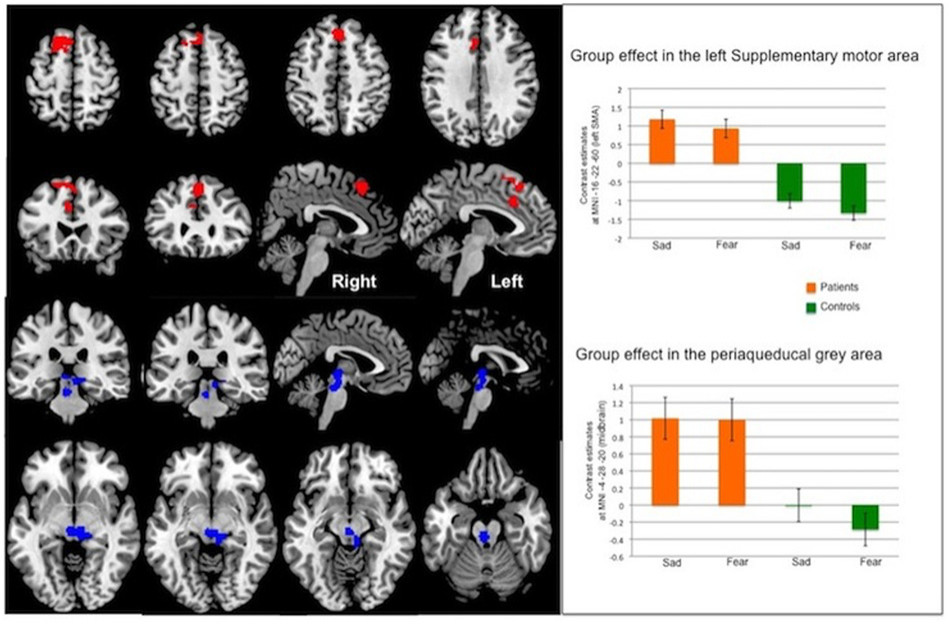
Whole-brain analysis: Group effect in the Emotion Model. Statistical maps have been thresholded at p<0.001 uncorrected. *Upper left panel*: Activation in a frontal cluster showing a significant group effect (CD patients > healthy controls). *Upper right panel*: Plots of beta estimates at MNI [–16 22 60] (left supplementary motor area).*Lower left panel*: Activation in a midbain cluster (including the periaqueducal grey area) showing a significant group effect (CD patients > healthy controls). *Lower right panel*: Plots of beta estimates at MNI [–4–28–20] (midbrain).

In the Emotion model, CD patients showed significant increased activity relative to healthy controls, in two clusters (Group effect): one in the midbrain comprising the peri-aqueductal gray (PAG) matter, and one in the frontal lobe; see Table 2 and Fig 3 overlay and plots in midbrain. The frontal cluster extended in the bilateral premotor/supplementary areas (Brodmann area 6), left dorsolateral prefrontal cortex (L DLPFC, Brodmann area 9) and left cingulate cortex (anterior BA 24 and medial BA 32). The peaks (see Table 2) were in the left superior frontal gyrus (premotor, BA6), left medial frontal gyrus (BA9) and left middle cingulate cortex (BA 24). The midbrain cluster included the periaqueducal grey area (see Fig 3). There was neither a valence effect nor an interaction (group X valence) effect.

No significant effects were found in the Linear-change model.

**Table 2:** Whole-brain analysis: Group effect (CD > controls) in the Emotion Model.

Cluster	Anatomical region (Brodmann Area)	pFWEcluster level	Cluster size	T value	Z score	MNI coordinates (x, y z)		
Midbrain	Midbrain (including periaqueductal grey area)	0.017	459	5.2	4.6	-4	-28	-20
				4.6	4.1	-2	-22	-2
				3.8	3.6	8	-32	-10
Frontal	L superior frontal gyrus (including the SMA)	0.010	528	4.7	4.2	-16	22	60
	L superior medial gyrus			4.4	4.0	2	30	52
	L middle cingulate cortex (BA 24)			3.9	3.6	-5	22	34

SMA: supplementary motor area

## Discussion

This study provides evidence, from ROI analyses, of increased activity of the amygdala in CD patients compared to controls when implicitly processing negative emotions—both fear and sadness. The behavioural data are consistent with this finding as both emotional conditions elicited better performance than neutral, suggesting increased attention-arousal for emotional stimuli. Furthermore the ROI analyses suggest that CD patients in comparison to controls may become progressively hypersensitive to fearful faces with repeated exposure, again implying dysfunctional emotion regulation. The whole-brain analysis revealed two highly significant clusters where patients had increased activity compared to healthy controls, which include the midbrain and superior frontal regions.

Our finding of increased amygdala activation in CD patients is consistent with several other studies finding either activation or connectivity changes in the amygdala and therefore adds further evidence for the association of emotion processing abnormalities with the disorder. A previous study[5] on emotional responses to fearful and happy faces revealed than, unlike healthy controls who have heightened amygdala activity during fear stimuli compared to happy stimuli, patients suffering from conversion disorder had similarly high responses for both emotions, suggesting an overall hyperarousal state when dealing with emotional stimuli. As mentioned in the introduction we have recently reported enhanced connectivity between the amygdala and the SMA in an emotional memory task providing a link between areas involved in emotional and motor control.[4] A similar limbic-motor interaction has been shown during an implicit emotion task similar to the present study with increased amygdala and SMA functional connectivity along with some evidence of a failure to habituate to the emotional stimulus[5]. Interestingly, a failure to habituate to aversive stimuli has been long recognised as a feature of ‘hysteria’ since the late 19^th^ century and has been replicated in behavioral studies[22]. Our data adds further information by showing that this hyperarousal state seems to me more specific for fear stimului, who convey a social threat dimension rather that any negative stimuli, as sad faces did not show this pattern over time.

Increased amygdala activity has also been found in CD patients using both internally and externally generated action selection tasks in movement disorder patients[23], and a case of CD with mutism[24] showed abnormal connectivity between the amygdala and motor (speech) centres. Alongside these clinical data there is evidence that the amygdala mediates fear-induced motor responses in healthy individuals[8] which explains the necessary presence of connections between the amygdala and motor systems in terms of an adaptive behavioural response to threat which could be postulated to be dysfunctional in CD. Finally the progressive sensitization to fear in the amygdala might be relevant to the clinical finding of persistent or recurrent somatic responses to stress and threat leading to enduring symptoms.

The periaqueductal grey (PAG) matter finding in the midbrain in our study is of particular interest as there is robust evidence from animal models for this being a key region in the ‘freeze response’ to threat, particularly via its connections with the amygdala[25]. There is also more recent evidence for the importance of the PAG in human responses to fear including autonomic ‘fear bradycardia’ responses[26]. In this context threat can range from complex stimuli (e.g. any modality of sensory information indicating the presence of a predator) to simple stimuli such as pain[27]. The PAG is established as a key region in pain pathways[28], and is thought to be involved in complex clinical pain syndromes such as migraine[29]. Therefore our findings of increased activation in CD patients to negative stimuli, suggest that triggering of an analogue of the freeze response might be involved in CD. Indeed, the potential role for dysfunction of the freeze response has previously been suggested as a biologically plausible mechanism for CD, [30]. Further studies, however, will be needed to replicate these findings in CD patients together with measurements of behavioural data (motor freezing and bradycardia) in order to definitely establish a link between abnormal freeze response in this disorder and increased PAG activity.

The cluster of increased activation we found in the frontal lobes of patients has its peak activation in the superior frontal gyrus which is an area thought to play a role in self-awareness, action monitoring and attention[31], which are cognitive processes with potential relevance to CD[3]. The cluster of activation also extends into several other medial frontal regions including middle frontal and premotor cortices, the SMA, and both anterior and middle cingulate gyrus. Whilst this is clearly a large range of structures many are united, to varying degrees, by their role in motor planning. As detailed above the SMA has been already been implicated as connecting emotion and motor symptoms in CD from experiments using both emotion tasks[4],[5] and purely motor tasks which nonetheless found links to emotion processing areas such as the amygdala and posterior cingulate[23]. Another study in CD also found a similar connection, this time with increased sensorimotor cortex activation alongside limbic regions including the anterior cingulate[32]. Differential cingulate activations have also been found in CD in a wide range of other studies.[24,33–40]. Finally a link between premotor regions and midbrain structures may play an important role in top-down control of motor defence mechanisms as discussed above. Anatomical connectivity has been shown in animals[41] and future studies should aim to also explore prefrontal motor cortex and PAG connectivity in CD.

Several limitations to this study should be noted. Firstly the generalizability of the results could be limited by the fact that the CD patients recruited into this study were mostly from specialist neuropsychiatry services and were therefore relatively severe cases. However, as symptom duration of more than 2 years was an exclusion criterion the patients had not had particularly chronic symptoms. Another potential limit to generalizability was that this study is only of motor CD patients, although there is no strong evidence for different mechanisms determining different neurological symptoms in CD. The specificity of these findings to CD can also be challenged as there was no psychiatric control group. However, the potential role of both anxiety and depression as confounders was addressed statistically and furthermore results stayed significant when corrected for anxiety. In fact, a recent large fMRI study of patients with anxiety and/or depression found that anxiety was associated with increased amygdala activation compared to controls but that the ‘shape’ of the response (including time-course) was not different[42]. Hence we propose that the pattern of physiological sensitization or non-habituation to fear in the amygdala may be a somewhat specific biomarker for the disorder. Another limitation is that we did not record clinical data on pain symptoms which often coexist in motor CD and this could be a confounding factor, particularly for the findings in the PAG given the evidence described above for the role of this area in nociceptive networks. Finally, this study is unable to address whether these differences in brain activation reflect the patient’s current state or whether it is a longer term trait and repeated scanning sessions, particularly investigating changes with symptomatic state would be required to answer this question.

In conclusion we have provided further evidence that patients with CD have abnormal emotion regulation with a progressive sensitization of the left amygdala with repeated exposure to negatively valenced emotional stimuli. Negative emotions in CD seems to also implicate motor circuits, such as the midbrain (PAG) and frontal structures (including SMA) that could reflect an abnormal freeze response to negative, including threatening stimuli.

## Supporting Information

S1 Fig Graphical representation of the regressors encoding the linear change in response to emotional stimuli over time, scaled by the relevant group-level beta coefficient. x axis = time in seconds, y axis = predicted BOLD signal.
(TIF)
