# Supplementary figures and images for: Emotion-Motion Interactions in Conversion Disorder: An fMRI Study
Source: PLoS One. 2015 Apr 10;10(4):e0123273. doi: 10.1371/journal.pone.0123273 (PMC4393246; doi:10.1371/journal.pone.0123273)

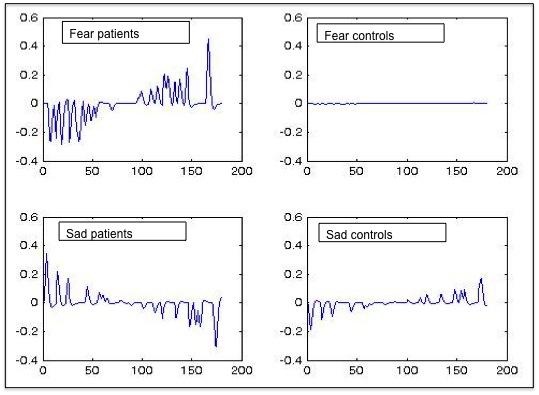

Supplement: S1 Fig — x axis = time in seconds, y axis = predicted BOLD signal. (TIF) [file pone.0123273.s001.tif]
